# Occupational risk factors for thumb carpometacarpal joint osteoarthritis: a register-based study of construction workers

**DOI:** 10.1136/oemed-2024-109949

**Published:** 2025-02-04

**Authors:** Charlotte Lewis, Jennie A Jackson, Albin Stjernbrandt, Gustav Andersson, Sebastian Mukka, Jens Wahlström, Per Liv

**Affiliations:** 1 Department of Public Health and Clinical Medicine, Umeå University, Umeå, Sweden; 2 Department of Occupational Health, Psychology and Sports Sciences, Faculty of Health and Occupational Studies, University of Gävle, Gävle, Sweden; 3 Department of Medical and Translational Biology, Umeå University, Umeå, Sweden; 4 Department of Diagnostics and Intervention, Umeå University, Umeå, Sweden

**Keywords:** Occupational Health, Vibration, Ergonomics, Osteoarthritis, Workload

## Abstract

**Objectives:**

The study investigated the association between occupational biomechanical risk factors and the occurrence of thumb carpometacarpal joint osteoarthritis (CMC1 OA) in construction workers.

**Methods:**

Male construction workers (n=237 525), participating in a Swedish occupational surveillance programme between 1971 and 1993, were followed between 1997 and 2019. CMC1 OA diagnoses were identified through linkage with national medical registries. Job title, smoking status, height, weight and age were collected from the surveillance programme. A job exposure matrix (JEM) was developed with exposure estimates on biomechanical risk factors for each occupational group. Relative risk (RR) of CMC1 OA diagnosis was calculated using a Poisson regression model.

**Results:**

There was an increased risk of CMC1 OA for all biomechanical risk factors (RR range 1.3–1.5). Exposure-response patterns were seen for repetitive wrist flexion and extension (low: RR 1.30 (95% CI 1.07 to 1.59), moderate: 1.32 (95% CI 1.07 to 1.62), high: 1.45 (95% CI 1.19 to 1.75)), wrist extension (low: 1.31 (95% CI 1.09 to 1.59), moderate: 1.41 (95% CI 1.17 to 1.70) and heavy lifting (low: 1.13 (95% CI 0.92 to 1.38), moderate: 1.45 (95% CI 1.18 to 1.77), high: 1.50 (95% CI 1.24 to 1.82). Electricians (1.29 (95% CI 1.03 to 1.89)), concrete workers (1.31 (95% CI 1.02 to 1.67)), plumbers (1.37 (95% CI 1.07 to 1.76)), sheet-metal workers (1.58 (95% CI 1.18 to 2.10)), wood workers (1.66 (95% CI 1.36 to 2.03)), repairers (1.75 (95% CI 1.06 to 2.90)) and glass workers (2.21 (95% CI 1.42 to 3.44) had an increased risk of CMC1 OA compared with the reference group.

**Conclusion:**

Wrist movements and hand loading were associated with CMC1 OA.

**WHAT IS ALREADY KNOWN ON THIS TOPIC**

- A few studies have suggested that work involving pinch grip, high physical load and repetitive movements increases the risk of developing carpometacarpal (CMC1) osteoarthritis (OA). However, the evidence is still insufficient, and there is a lack of larger, longitudinal studies.

**WHAT THIS STUDY ADDS**

- This study adds to the sparse literature on an association between occupational biomechanical exposure and CMC1 OA.

**HOW THIS STUDY MIGHT AFFECT RESEARCH, PRACTICE OR POLICY**

- The results show an increased risk of developing CMC1 OA for work involving high grip force, pinch grip, repetitive wrist movements, hand-held tool use, heavy lifting and hand-arm vibration and highlight a possible potential of preventing CMC1 OA by reducing these exposures.

## Introduction

 Osteoarthritis (OA), the most common joint disease in the world, can lead to disability for those affected and burden healthcare systems.^1^ Every synovial joint in the body can be affected by OA, but it is most common in the knee, hip, spine and hand.^2^ Within the hand, the joint most often affected by OA is the thumb carpometacarpal (CMC) joint.^3^ CMC joint OA of the thumb (CMC1 OA) is typically characterised by stiffness, pain with grip and pinch, and aches.^4 5^ The disease is caused by degradation of the articular cartilage in combination with inflammation in the joint capsule and synovia, subchondral sclerosis and formation of osteophytes.^6^ Clinical findings such as point tenderness at the CMC joint, and pain with axial grinding of the metacarpal bone on the trapezium are characteristic of CMC1 OA. Pinch strength is almost always diminished and functional hand width is narrowed.^7^ Radiographic imaging of the joint can be used to confirm the diagnosis and radiographically stage the disease, as described by Eaton.^7^ However, it has been presented that only 28% of women who have radiographic features of CMC1 OA report actual symptoms, highlighting the need to combine radiographic imaging with patient history and clinical examination.^8^ Non-operative treatment consists of splinting, corticoid injections and occupational therapy. When conservative treatment is not sufficient, operative treatment, such as trapeziectomy, with or without tendon interposition, is performed. Other commonly used techniques include arthrodesis, selective CMC1 denervation or total joint replacement.^9^,^14^ The choice of surgical technique often depends on the surgeon’s preference, and the presence of comorbidities such as thumb metacarpophalangeal (MCP1) hypermobility or scaphotrapeziotrapezoid joint OA, where additional procedures such as MCP1 arthrodesis or partial trapezoid excision are commonly performed, respectively.^15^

Although genetic, metabolic and inflammatory factors have been described,^6^ advanced age is the most pronounced risk factor for the development of CMC1 OA.^16 17^ Female gender is another undisputed risk factor,^316^,^20^ where an OR of 1.30 (95% CI 1.05 to 1.61) has been presented for females compared with males.^17^ This gender difference is seen in all age brackets.^17^ Additionally, body mass index (BMI) has been suggested as a risk factor.^19^ Biomechanical and occupational risk factors for CMC1 OA were considered already in the late 1950s when Kellgren and Lawrence defined radiological criteria and described the prevalence among different occupational groups in the general population.^21^ Male cotton workers had a higher prevalence of CMC1 OA compared with other occupational groups. The findings were subsequently more thoroughly described and occupational biomechanical exposure was put forward as a plausible mechanism.^22^ Later studies have reported an association between occupational load, such as repetitive thumb use, work involving pinch grip and high physical load.^23^,^25^ However, there is yet insufficient evidence to determine in detail what occupational biomechanical factors increase the risk of CMC1 OA. The aim of our study was to evaluate the association between occupational biomechanical exposures and occurrence of CMC1 OA.

## Methods

### Study design and setting

This register-based cohort study followed Swedish workers in the construction industry who had participated in a national occupational health surveillance programme (Bygghälsan). The total cohort consisted of 389 132 individuals who participated in at least one health examination between 1971 and 1993. While participation was voluntary, about 80% of eligible workers participated in the health surveillance programme.

### Participants

**Figure 1:**
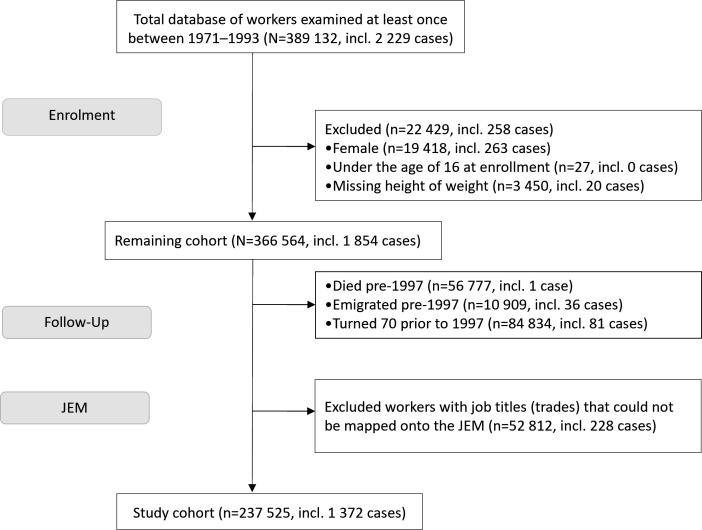
Flow diagram outlining exclusions from total construction worker cohort and resulting study cohort. JEM, job exposure matrix.

Only male construction workers were included in the current study since only 5.3% of the cohort were female, of which 69% could not be included in the job exposure matrix (JEM) due to lacking data on occupational group, and of the remaining women, 85% were employed in white-collar positions and thus would be considered reference group workers. Workers who were less than 16 years old at their first health examination, or died, emigrated, or retired prior to follow-up were excluded from analyses. Workers for whom no job title was recorded at any of the medical examinations or who were classified in the non-specific ‘other’ work group were also excluded since no exposure could be assigned to them using the JEM. The remaining workers (N=237 525) comprised the study cohort. Data on deaths and emigration among the workers were obtained through linkage with the Total Population Register, held by Statistics Sweden. Data on what years the participants were employed in the construction industry were retrieved from the Swedish Longitudinal Integrated Database for Health Insurance and Labour Market Studies.^26^ A detailed description of the number of workers (including cases) excluded at each level is presented in figure 1.

### Case definition

The case definition in the present study was defined as diagnose by physician, which could reflect clinical or radiological diagnosis. We identified cases from the national hospital and outpatient registries who had at least once been assigned any of the following International Statistical Classification of Diseases and Related Health Problems 10th edition (ICD-10) codes: M18.1 (Primary CMC1 OA, unilateral or without further specification), M18.3 post-traumatic CMC1 OA, unilateral or without further specification), M18.5 (other secondary CMC1 OA, unilateral or without further specification) or M18.9 (CMC1 OA, unspecified). The data retrieval period spanned from 1 January 1997 to 31 December 2019. Register linkage was achieved using the unique personal identification number assigned to each Swedish resident with computerised inpatient and outpatient data. For the distribution of the diagnostic codes, see online supplemental table S1.

### Individual factors

Self-reported worker height, weight and age were obtained from the first examination in the surveillance programme at which they were reported, while smoking status was obtained from the last examination at which it was reported. Workers were categorised into current smoker, ex-smoker, never smoker and unknown smoking status. BMI was calculated and classified as underweight (BMI<18.5 kg/m^2^), normal (18.5≤BMI<25 kg/m^2^), overweight (25≤BMI<30 kg/m^2^) or obese (BMI≥30 kg/m^2^).

### Biomechanical exposure

The job title was obtained from the last examination at which it was reported and coded using occupational work codes applied in the Swedish construction industry at the time of the health examination. Prior to 1986, 212 individual job title codes were used, while 90 job title codes were used from 1986 onwards. The job titles were mapped to 21 occupational groups developed by technical experts of the industry to group workers performing similar work tasks and having similar training. Biomechanical exposure in each occupational group was categorised in a JEM developed for the construction worker cohort and previously used to assess occupational risk factors for other health outcomes.^27^,^29^ Based on the available literature, eight JEM exposures were considered relevant to assess occupational risk factors for CMC1 OA and were included in the current study. The variables were ‘magnitude of handgrip force’, ‘frequency of pinch grip’, ‘frequency of repetitive wrist flexion and extension work’, ‘frequency of full wrist extension’, ‘frequency of hand-held tool use’, ‘frequency of heavy lifting’, ‘magnitude of hand-arm vibration (HAV)’ and ‘frequency of impact shocks during HAV’. The biomechanical exposure variables have previously been described in detail except for ‘frequency of pinch grip’ that was added for the current paper.^28^ See online supplemental table S2 for variables and JEM ratings for each occupational group.

### Statistical analysis

For each worker, the number of person-years at risk was calculated from the start of the follow-up period (1 January 1997) until the date of becoming a case, emigration, death or end of the follow-up period (31 December 2019). The overall incidence was calculated with a 95% CI. The follow-up time of each worker was split into 1-year intervals to account for age and calendar time effects. Assuming year-wise constant incidence, robust Poisson regression model was used to estimate relative risks (RRs) of CMC1 OA for JEM levels, comparing occupations with low, moderate and high exposure to a reference group of white-collar workers and foremen. Models were adjusted for age, smoking status (all categories), BMI (continuously) and calendar time. Effects of age, height, BMI and calendar time were modelled using natural cubic splines with three knots placed at the 10th, 50th and 90th percentile of the corresponding variable distribution. Robust Hubert-White SEs were used to estimate CIs.

## Results

The study cohort (n=237 525) accumulated a total number of 4 233 388 person-years of observation including 1372 cases of CMC1 OA. For the distribution of cases for each diagnostic code, see online supplemental table S1. The 23-year incidence rate (IR) of CMC1 OA was 32.4 cases per 100 000 person-years.

Analysis did not show an association between height and CMC1 OA. However, an increased risk was seen among those who were current or ex-smokers, RR 1.70 (95% CI 1.52 to 1.90) and those categorised as overweight, RR 1.24 (95% CI 1.10 to 1.40) (table 1). The IR of CMC1 OA increased with age, with a large increase starting around age 40 (online supplemental figure S1). The IR was also affected by date of diagnosis with peak rates occurring around 2010 (online supplemental figure S2). Accordingly, biomechanical risk factor models were adjusted for age, BMI, smoking status and calendar time. Results from unadjusted analyses, in accordance with Strengthening the Reporting of Observational Studies in Epidemiology reporting guidelines, are reported in online supplemental table S3.

**Table 1:** Incidence rates (IRs) and relative risks (RRs) for CMC1 OA according to age, height, smoking habits and body mass index (BMI)

	N	Person-years	Cases	IR	RR	95% CI
Height (cm)						
<160	593	8539	1	112	0.35	0.05 to 2.49
160–170	25 105	392 724	134	34	1.02	0.85 to 1.23
170–180	125 494	2 183 976	730	33	1.00	Ref
180–190	79 025	1 499 766	465	31	0.93	0.83 to 1.04
190–200	7308	148 384	42	28	0.85	0.62 to 1.16
Smoking						
Never	104 048	2 017 542	487	24	1.00	Ref
Ever	121 340	2 015 017	827	41	1.70	1.52 to 1.90
Unknown	12 137	200 830	58	29	1.20	0.54 to 0.92
BMI						
Underweight	3667	76 688	16	21	0.68	0.41 to 1.11
Normal weight	162 919	3 044 417	936	31	1.00	Ref
Overweight	62 526	986 958	376	38	1.24	1.10 to 1.40
Obese	8413	125 326	44	35	1.14	0.84 to 1.54

RRs are presented as crude estimates.

OA, osteoarthritis.

There was an association between all biomechanical risk factors included in our analysis and the risk of CMC1 OA (table 2). Exposure to high handgrip force (RR 1.54, 95% CI 1.28 to 1.85), moderate frequency of pinch grip (RR 1.50, 95% CI 1.24 to 1.80), high frequency of heavy lifting (RR 1.50, 95% CI 1.24 to 1.82) and a moderate magnitude of HAV (RR 1.52, 95% CI 1.26 to 1.83) showed the highest RRs of around 1.5. A positive exposure-response pattern was suggested for frequency of repetitive wrist flexion and extension work, frequency of full wrist extension, frequency of handheld tool use and frequency of heavy lifting. Electricians (RR 1.29, 95% CI 1.03 to 1.89), concrete workers (RR 1.31, 95% CI 1.02 to 1.67), plumbers (RR 1.37, 95% CI 1.07 to 1.76), sheet-metal workers (RR 1.58, 95% CI 1.18 to 2.10), wood workers (RR 1.66, 95% CI 1.36 to 2.03), repairers (RR 1.75, 95% CI 1.06 to 2.90) and glass workers (RR 2.21, 95% CI 1.42 to 3.44) were at higher RR for CMC1 OA compared with the reference group, which comprised foremen and white-collar workers (table 3).

**Table 2:** Biomechanical risk factors and the incidence rate (IR) and relative risk (RR) for CMC1 OA in the study cohort of construction workers (N=237 525)

	N	Person-years	Cases	IR	RR	95% CI
Magnitude of handgrip force						
Reference	30 640	486 401	134	16	1.00	Ref
Low	18 189	292 992	88	30	1.04	0.80 to 1.37
Moderate	78 163	1 459 642	411	28	1.21	0.99 to 1.47
High	110 533	1 994 353	739	37	1.54	1.28 to 1.85
Frequency of pinch grip						
Reference	30 640	827 820	134	28	1.00	Ref
Low	21 111	350 316	101	29	1.04	0.80 to 1.34
Moderate	114 818	2 050 913	746	36	1.50	1.24 to 1.80
High	70 956	1 345 758	391	29	1.26	1.03 to 1.53
Frequency of repetitive wrist flexion and extension work						
Reference	30 640	486 401	134	28	1.00	Ref
Low	73 199	1 253 077	416	33	1.30	1.07 to 1.59
Moderate	51 976	981 574	302	31	1.32	1.07 to 1.62
High	81 710	1 512 337	520	34	1.45	1.19 to 1.75
Frequency of full wrist extension						
Reference	30 640	486 401	134	28	1.00	Ref
Low	99 801	1 758 419	566	32	1.31	1.09 to 1.59
Moderate	107 084	1 988 569	672	34	1.41	1.17 to 1.70
High	–	–	–	–	–	–
Frequency of hand-held tool use						
Reference	30 640	486 401	134	28	1.00	Ref
Low	20 527	336 493	111	33	1.17	0.91 to 1.51
Moderate	18 734	335 466	110	33	1.34	1.04 to 1.72
High	167 624	3 075 029	1 017	33	1.39	1.16 to 1.67
Frequency of heavy lifting						
Reference	30 640	486 401	134	28	1.00	Ref
Low	68 268	1 265 516	344	27	1.13	0.92 to 1.38
Moderate	49 841	884 115	319	36	1.45	1.18 to 1.77
High	88 776	1 597 357	575	36	1.50	1.24 to 1.82
Magnitude of hand-arm vibration (HAV)						
Reference	30 640	486 401	134	28	1.00	Ref
Low	76 291	1 331 856	393	30	1.17	0.96 to 1.43
Acceptable	103 355	1 979 867	697	35	1.52	1.26 to 1.83
High	27 239	435 265	148	34	1.34	1.06 to 1.70
Frequency of impact shocks during HAV						
Reference	30 640	486 401	134	28	1.00	–
Rare	136 094	2 461 789	824	33	1.37	1.14 to 1.64
Often	70 791	1 285 199	414	32	1.36	1.12 to 1.65

RRs were adjusted for body mass index, smoking, age and calendar time. The foremen and white-collar workers were used as reference.

OA, osteoarthritis.

**Table 3:** Incidence rates (IRs) and relative risks (RR) for CMC1 OA, adjusted for body mass index, smoking, age and calendar time according to occupational group

Occupational group	N	Person-years	Cases	IR	RR	95% CI
Foremen and white-collar workers	30 640	486 401	134	28	1.00	Ref
Heavy machinery operators	8938	147 228	32	22	0.75	0.51 to 1.10
Brick layers	7150	120 209	27	22	0.96	0.63 to 1.45
Insulators	2297	42 281	10	24	0.98	0.52 to 1.87
Painters	19 041	353 981	84	24	1.01	0.77 to 1.34
Asphalt workers	3268	55 542	18	32	1.20	0.73 to 1.97
Preparatory workers	8577	148 860	45	30	1.22	0.87 to 1.71
Rock workers	2115	30 037	11	37	1.26	0.68 to 2.33
Electricians	32 109	629 623	183	29	1.29	1.03 to 1.89
Concrete workers	22 991	369 133	120	33	1.31	1.02 to 1.67
Drivers	3368	53 362	21	39	1.34	0.85 to 2.13
Roofers	1129	20 363	7	34	1.34	0.62 to 2.86
Plumbers	19 867	351 950	119	34	1.37	1.07 to 1.76
Floor layers	4602	86 364	29	34	1.44	0.96 to 2.16
Crane operators	2615	36 860	17	46	1.53	0.92 to 2.54
Sheet-metal workers	10 318	198 445	74	37	1.58	1.18 to 2.10
Wood workers	52 904	1 001 287	392	39	1.66	1.36 to 2.03
Refrigerator technicians	1125	21 866	9	41	1.71	0.87 to 3.37
Repairers	2133	36 095	17	47	1.75	1.06 to 2.90
Glass workers	2338	43 500	23	53	2.21	1.42 to 3.44

Occupational groups are shown in order of ascending RR. The foremen and white-collar workers were used as reference.

OA, osteoarthritis.

## Discussion

In our cohort, age, ever-smoking and BMI were associated with CMC1 OA. The highest RRs were seen for exposure to high handgrip force (RR 1.54, 95% CI 1.28 to 1.85), moderate frequency of pinch grip (RR 1.50, 95% CI 1.24 to 1.80), high frequency of heavy lifting (RR 1.50, 95% CI 1.24 to 1.82) and a moderate magnitude of HAV (RR 1.52, 95% CI 1.26 to 1.83). Sheet-metal workers (RR 1.58, 95% CI 1.18 to 2.10), wood workers (RR 1.66, 95% CI 1.36 to 2.03), repairers (RR 1.75, 95% CI 1.06 to 2.90) and glass workers (RR 2.21, 95% CI 1.42 to 3.44) had the highest RRs for CMC1 OA compared to the references.

In accordance with our results on individual factors, a nationwide Swedish registry study presented a prevalence of physician-diagnosed CMC1 OA of 1.7% in 30–34 years compared with 3.6% in 65–69 years.^16^ Also, a recent meta-analysis including 16 studies suggested an OR of 1.06 (95% CI 1.06 to 1.07) per year of age for men and women combined.^17^ Further, a large Finnish population study presented an association between BMI and prevalence of CMC1 OA in both men and women, with an adjusted OR of 1.29 (95% CI 1.15 to 1.43) per 5 kg/m^2^ increment in BMI.^19^

Regarding occupational risk factors, an earlier case–control study including 61 women surgically treated for CMC1 OA and 120 age-matched controls found elevated risk with OR as high as 11.91 (95% CI 3.65 to 38.86) in occupations involving repetitive thumb use.^23^ The study also found increased OR in occupations for which the main tasks require thumb use with an OR of 3.78 (95% CI 1.20 to 11.92). Besides the subjects being women, the occupations were mainly secretaries, tailors, dressmakers, hatters, sewers, embroiderers, domestic helpers and cleaners. These are occupations with more repetitive and monotonous tasks compared with the construction occupations in the present study. Also, the case–control study had surgically treated CMC1 OA as case criteria. Described differences are possible explanations for the difference in magnitude of risk between the two studies.^23^ Similar to our results, a meta-analysis showed an association of pinch grip work with CMC1 OA, with an OR of 2.10 (95% CI 1.06 to 4.17).^24^ A case–control study using a Swedish healthcare register with 3462 physician-diagnosed CMC1 OA patients and 13 211 controls matched on age, sex, education level and postcode found increased risk of CMC1 OA in men performing light–moderate work (OR 1.31, 95% CI 0.96 to 1.79), moderate work (OR 1.76, 95% CI 1.29 to 2.40) and heavy work (OR 2.00, 95% CI 1.59 to 2.51) compared with the reference group of men performing light work.^25^ An increased risk was also present for women performing light–moderate work (OR 1.46, 95% CI 1.32 to 1.61), moderate work (OR 1.27, 95% CI 1.10 to 1.46) and heavy work (OR 1.31, 95% CI 1.07 to 1.59) compared with light work.^25^ The generalised level of exposure assessment (light, moderate and heavy) in the forementioned study complicates direct comparison with the findings of the current study but also suggest that physical load is a risk factor for CMC1 OA.

Contrary to our results, the previously mentioned meta-analysis did not show an association between CMC1 OA and handgrip work or exposure to HAV.^24^ Some of the studies included in the meta-analysis had different study populations compared with the present study, for example, considering only women, as well as including different occupations, such as secretaries, tailors, dressmakers and hatters where hand grip and/or HAV exposures may have been considerably lower than in our construction cohort. Also, the meta-analysis defined cases based on radiographic classification and/or surgical treatment, while the present study defined cases based only on diagnosis. Regarding the studies on HAV, the average age of the participants included in the meta-analysis was 34 to 46 years old, which is quite young compared with our study. Further, most studies had a reference group that comprised manual workers who were also likely to have had exposure to the investigated biomechanical exposure factors.

Previous studies have suggested that one key pathomechanism for OA involves degenerative changes in the articular cartilage triggered by long-term high mechanical loading.^30 31^ High loads in the CMC1 joint during both pinch grip and grasp have been documented,^32^ and our findings of increased OA risk with pinch grip and heavy loading further support this mechanism. Although previous evidence regarding the role of HAV has been contradictory, exposure to impacts associated with vibrating machinery has been proposed as one exposure factor of importance.^33^ Possible mechanisms of vibration-induced OA include repeated microtrauma to cartilage and subchondral bone, altered mechanical signalling in chondrocytes, compromised microvascular circulation, abnormal bone remodelling and upregulated inflammatory pathways; however, definitive scientific evidence for these theories is still lacking.

In our analysis, the occupations with the highest risk were sheet-metal workers, wood workers, maintenance/repair workers and glassworkers. Sheet-metal workers and glassworkers frequently handle materials requiring strong pinch grip, while woodworkers and repairers are likely to be exposed to repetitive hand movements, grip force, HAV and pinch grip.

A JEM is often considered one of the best available methods for retrospective exposure assessment in cohort studies.^34^ In our study, the JEM was based on historical records including detailed descriptions of tasks within each job title as well as biomechanical exposures. However, it was not validated against any technical measurement. Further, a JEM does not consider variation between workers within a job title since the exposures are assigned at a group level, nor can it take into account that exposure in an occupational group can change over time. Also, there is a possibility of healthy worker effect, where subjects with CMC1 OA would have left the construction industry, leading to an underestimation of the risk. However, we conducted a supplementary analysis on a subset of workers still in construction trade for at least 1 year during a 5-year period prior to our follow-up period which showed similar results in risk estimates; this argues against a healthy worker effect (online supplemental table S4). The fact that the biomechanical risk factors in the JEM were correlated makes it difficult to speculate on specific pathomechanistic explanations related to specific exposure factors. Lastly, the JEM used in the present study did not assess cumulative exposure of the biomechanical exposures.

We opted out of using an even stricter case definition of surgically treated CMC1 OA since it is common to wait until after retirement to proceed with surgery, due to the functional impairment (reduced mobility) that comes with arthrodesis, a commonly used surgery technique. Therefore, we would risk missing out on workers having their surgery at an older age. The decision to include the diagnose codes M18.3 (post-traumatic CMC1 OA) and M18.5 (other secondary CMC1 OA), even though they would not be caused by occupational exposure, was due to clinical experience of misclassification in reporting different subdiagnoses. There were only a few cases of M18.3 and M18.5 (n=62), and a sensitivity analysis performed excluding those two diagnosis codes from the case definition showed similar results (online supplemental table S5). White-collar workers and foremen from the same cohort comprised the reference group. They were considered suitable since they were employed within the same organisation, but in general, had no heavy physical exposure at work. There is, however, a risk that the foremen could have previously worked in a construction trade, and therefore, have had earlier exposure which could lead to underestimation of the risk for the occupational groups compared with a completely unexposed group. This study included only men, and even though it would be reasonable to assume associations for women as well, direct generalisation cannot be made.

This study adds to the sparse literature on the associations between occupational biomechanical exposure and CMC1 OA. The results highlight a possible potential of preventing CMC1 OA by reducing handloading, repetitive wrist movements and exposure to HAV.

## Supplementary material

10.1136/oemed-2024-109949 online supplemental file 1

## Data Availability

Data are available on reasonable request.
