# Supplementary material for: Occupational risk factors for thumb carpometacarpal joint osteoarthritis: a register-based study of construction workers
Source: Occup Environ Med. 2025 Feb 4;82(1):e109949. doi: 10.1136/oemed-2024-109949 (PMC12015016; doi:10.1136/oemed-2024-109949)
Supplement: online supplemental file 1 [file oemed-82-1-s001.docx]

| **Table S1.** Distribution of diagnostic codes (10th International Classification of Diseases), of included cases, N (%). | | |
| --- | --- | --- |
| Diagnose code |  | N (%) |
| M18.1 | Primary CMC1 OA, unilateral or unspecified | 972 (70.8) |
| M18.3 | Posttraumatic CMC1 OA, unilateral or unspecified | 34 (2.5) |
| M18.5 | Other secondary CMC1 OA, unilateral or unspecified | 28 (2.0) |
| M18.9 | CMC1 OA, unspecified | 338 (24.6) |

| **Table S2.** Job exposure matrix mapping for all included occupational groups. | | | | | | | | | | | | | | | |  |
| --- | --- | --- | --- | --- | --- | --- | --- | --- | --- | --- | --- | --- | --- | --- | --- | --- |
| Occupational group | | *Magnitude of handgrip force^a^* | *Frequency of pinch grip use^a^* | | *Frequency of repetitive wrist flexion and extension work^a^* | | *Frequency of full wrist extension^a^* | *Frequency of hand-held tool use^a^* | | *Frequency of heavy lifting (>25 kg)^a^* | | *Magnitude of hand-arm vibration (HAV)^b^* | | | *Frequency of impact shocks during HAV^c^* |  |
| Asphalt workers (1) | | 1 | 1 | | 1 | | 1 | 1 | | 1 | | 1 | | | 1 |  |
| Rock workers (2) | | 3 | 1 | | 1 | | 1 | 3 | | 2 | | 3 | | | 3 |  |
| Concrete workers (3) | | 3 | 2 | | 1 | | 1 | 3 | | 2 | | 3 | | | 3 |  |
| Wood workers (4) | | 3 | 2 | | 3 | | 2 | 3 | | 2 | | 2 | | | 1 |  |
| Brick layers (5) | | 2 | 3 | | 3 | | 2 | 3 | | 2 | | 1 | | | 1 |  |
| Floor layers (6) | | 2 | 2 | | 1 | | 1 | 2 | | 2 | | 2 | | | 1 |  |
| Heavy machinery operators (7) | | 1 | 1 | | 1 | | 2 | 1 | | 1 | | 1 | | | 1 |  |
| Crane operators (8) | | 1 | 2 | | 3 | | 2 | 1 | | 1 | | 1 | | | 1 |  |
| Drivers (9) | | 1 | 1 | | 1 | | 2 | 1 | | 2 | | 1 | | | 1 |  |
| Glass workers (10) | | 3 | 3 | | 1 | | 1 | 1 | | 2 | | 1 | | | 1 |  |
| Insulators (11) | | 2 | 1 | | 1 | | 1 | 2 | | 1 | | 2 | | | 1 |  |
| Refrigerator technicians (12) | | 2 | 1 | | 1 | | 1 | 2 | | 2 | | 2 | | | 3 |  |
| Plumbers (13) | | 3 | 2 | | 2 | | 1 | 3 | | 2 | | 1 | | | 1 |  |
| Painters (14) | | 2 | 3 | | 3 | | 1 | 3 | | 2 | | 1 | | | 1 |  |
| Sheet-metal workers (15) | | 3 | 3 | | 1 | | 1 | 3 | | 2 | | 2 | | | 3 |  |
| Electricians (16) | | 2 | 3 | | 2 | | 2 | 3 | | 1 | | 2 | | | 3 |  |
| Foremen (17) | | ref | ref | | ref | | ref | ref | | ref | | ref | | | ref |  |
| White collar workers (18) | | ref | ref | | ref | | ref | ref | | ref | | ref | | | ref |  |
| Repairers (19) | | 2 | 2 | | 1 | | 1 | 2 | | 2 | | 3 | | | 3 |  |
| Preparatory workers (20) | | 2 | 2 | | 1 | | 1 | 2 | | 2 | | 1 | | | 1 |  |
| Roofers (21) | | 2 | 2 | | 1 | | 1 | 3 | | 3 | | 1 | | | 1 |  |
| a. 1 = low, 2 = moderate, 3 = high  b. 1 = none, 2 = acceptable, 3 = high  c. 1 = rare, 3 = often | | | | | | | | | | | | | | | |  |
| **Table S3.** Biomechanical risk factors and the incidence rate (IR) and relative risk (RR) for CMC1 OA in the study cohort of construction workers (N=237 525), unadjusted. The foremen and white-collar workers were used as reference. | | | | | | | | | | | | | | | |  |
|  | | | N | | person–years | | | cases | | IR | | RR | 95% CI | | |  |
| *Magnitude of handgrip force* | | | | | | | | | | | | | | | |  |
| Reference | | | 30 640 | | 486 401 | | | 134 | | 28 | | 1.00 | Ref | | |  |
| Low | | | 18 189 | | 292 992 | | | 88 | | 30 | | 1.09 | 0.83–1.43 | | |  |
| Moderate | | | 78 163 | | 1 459 642 | | | 411 | | 28 | | 1.02 | 0.84–1.24 | | |  |
| High | | | 110 533 | | 1 994 353 | | | 739 | | 37 | | 1.35 | 1.12–1.62 | | |  |
| *Frequency of pinch grip* | | | | | | | | | | | | | | | |  |
| Reference | | | 30 640 | | 486 401 | | | 134 | | 28 | | 1.00 | Ref | | |  |
| Low | | | 21 111 | | 350 316 | | | 101 | | 29 | | 1.05 | 0.81–1.35 | | |  |
| Moderate | | | 114 818 | | 2 050 913 | | | 746 | | 36 | | 1.32 | 1.10–1.59 | | |  |
| High | | | 70 956 | | 1 345 758 | | | 391 | | 29 | | 1.05 | 0.87–1.28 | | |  |
| *Frequency of repetitive wrist flexion and extension work* | | | | | | | | | | | | | | | |  |
| Reference | | | 30 640 | | 486 401 | | | 134 | | 28 | | 1.00 | Ref | | |  |
| Low | | | 73 199 | | 1 253 077 | | | 416 | | 33 | | 1.21 | 0.99–1.46 | | |  |
| Moderate | | | 51 976 | | 981 574 | | | 302 | | 31 | | 1.12 | 0.91–1.37 | | |  |
| High | | | 81 710 | | 1 512 337 | | | 520 | | 34 | | 1.25 | 1.03–1.51 | | |  |
| *Frequency of full wrist extension* | | | | | | | | | | | | | | | |  |
| Reference | | | 30 640 | | 486 401 | | | 134 | | 28 | | 1.00 | Ref | | |  |
| Low | | | 99 801 | | 1 758 419 | | | 566 | | 32 | | 1.17 | 0.97–1.41 | | |  |
| Moderate | | | 107 084 | | 1 988 569 | | | 672 | | 34 | | 1.23 | 1.02–1.48 | | |  |
| High | | | - | | - | | | - | | - | | - | - | | |  |
| *Frequency of hand–held tool use* | | | | | | | | | | | | | | | |  |
| Reference | | | 30 640 | | 486 401 | | | 134 | | 28 | | 1.00 | Ref | | |  |
| Low | | | 20 527 | | 336 493 | | | 111 | | 33 | | 1.20 | 0.93–1.54 | | |  |
| Moderate | | | 18 734 | | 335 466 | | | 110 | | 33 | | 1.19 | 0.93–1.53 | | |  |
| High | | | 167 624 | | 3 075 029 | | | 1017 | | 33 | | 1.20 | 1.00–1.44 | | |  |
| *Frequency of heavy lifting* | | | | | | | | | | | | | | | |  |
| Reference | | | 30 640 | | 486 401 | | | 134 | | 28 | | 1.00 | Ref | | |  |
| Low | | | 68 268 | | 1 265 516 | | | 344 | | 27 | | 0.99 | 0.81–1.20 | | |  |
| Moderate | | | 49 841 | | 884 115 | | | 319 | | 36 | | 1.31 | 1.07–1.60 | | |  |
| High | | | 88 776 | | 1 597 357 | | | 575 | | 36 | | 1.31 | 1.08–1.58 | | |  |
| *Magnitude of hand–arm vibration (HAV)* | | | | | | | | | | | | | | | |  |
| Reference | | | 30 640 | | 486 401 | | | 134 | | 28 | | 1.00 | Ref | | |  |
| Low | | | 76 291 | | 1 331 856 | | | 393 | | 30 | | 1.07 | 0.88–1.33 | | |  |
| Acceptable | | | 103 355 | | 1 979 867 | | | 697 | | 35 | | 1.28 | 1.06–1.54 | | |  |
| High | | | 27 239 | | 435 265 | | | 148 | | 34 | | 1.23 | 0.98–1.56 | | |  |
| *Frequency of impact shocks during HAV* | | | | | | | | | | | | | | | |  |
| Reference | | | 30 640 | | 486 401 | | | 134 | | 28 | | 1.00 | Ref | | |  |
| Rare | | | 136 094 | | 2 461 789 | | | 824 | | 33 | | 1.21 | 1.01–1.46 | | |  |
| Often | | | 70 791 | | 128 520 | | | 414 | | 32 | | 1.17 | 0.96–1.42 | | |  |
| *N – number workers; IR – incidence rate per 100 000 person–years; RR – relative risk; CI – confidence interval* | | | | | | | | | | | | | | | |  |
| **Table S4**. Biomechanical risk factors and the incidence rate (IR) and relative risk (RR) for CMC1 OA in workers still employed in construction trade at least one years within the 5-year period prior to follow up (N= 155 723). RR were adjusted for BMI, smoking, age, and calendar time of surgery. The foremen and white-collar workers were used as reference. | | | | | | | | | | | | | | | |  |
|  | | | N | | person-years | | | cases | | IR | | RR | 95% CI | | | |
| *Magnitude of handgrip force* | | | | | | | | | | | | | | | | |
| Reference | | | 18 806 | | 307 167 | | | 87 | | 28 | | 1.00 | Ref | | | |
| Low | | | 10 749 | | 182 329 | | | 55 | | 30 | | 1.01 | 0.72–1.42 | | | |
| Moderate | | | 52 285 | | 1 014 930 | | | 290 | | 29 | | 1.21 | 0.95–1.55 | | | |
| High | | | 73 883 | | 1 405 683 | | | 531 | | 38 | | 1.54 | 1.22–1.93 | | | |
| *Frequency of pinch grip* | | | | | | | | | | | | | | | | |
| Reference | | | 18 806 | | 307 167 | | | 87 | | 28 | | 1.00 | Ref | | | |
| Low | | | 12 793 | | 223 695 | | | 66 | | 30 | | 1.02 | 0.74–1.41 | | | |
| Moderate | | | 76 211 | | 1 440 493 | | | 529 | | 37 | | 1.48 | 1.18–1.86 | | | |
| High | | | 47 913 | | 938 753 | | | 281 | | 30 | | 1.29 | 1.01–1.64 | | | |
| *Frequency of repetitive wrist flexion and extension work* | | | | | | | | | | | | | | | | |
| Reference | | | 18 806 | | 307 167 | | | 87 | | 28 | | 1.00 | Ref | | | |
| Low | | | 45 001 | | 815 819 | | | 276 | | 34 | | 1.29 | 1.01–1.65 | | | |
| Moderate | | | 34 257 | | 670 257 | | | 204 | | 30 | | 1.30 | 1.01–1.68 | | | |
| High | | | 57 659 | | 1 116 865 | | | 396 | | 35 | | 1.46 | 1.16–1.85 | | | |
| *Frequency of full wrist extension* | | | | | | | | | | | | | | | | |
| Reference | | | 18 806 | | 307 167 | | | 87 | | 28 | | 1.00 | Ref | | | |
| Low | | | 64 929 | | 1 208 770 | | | 386 | | 32 | | 1.27 | 1.01–1.61 | | | |
| Moderate | | | 71 988 | | 1 394 172 | | | 490 | | 35 | | 1.45 | 1.15–1.82 | | | |
| High | | | - | | - | | | - | | - | | - | - | | | |
| *Frequency of hand-held tool use* | | | | | | | | | | | | | | | | |
| Reference | | | 18 806 | | 307 167 | | | 87 | | 28 | | 1.00 | Ref | | | |
| Low | | | 12 196 | | 209 555 | | | 73 | | 35 | | 1.19 | 0.87–1.63 | | | |
| Moderate | | | 11 869 | | 224 231 | | | 73 | | 33 | | 1.29 | 0.95–1.77 | | | |
| High | | | 112 852 | | 2 169 157 | | | 730 | | 34 | | 1.40 | 1.12–1.75 | | | |
| *Frequency of heavy lifting* | | | | | | | | | | | | | | | | |
| Reference | | | 18 806 | | 307 167 | | | 87 | | 28 | | 1.00 | Ref | | | |
| Low | | | 45 272 | | 869 968 | | | 243 | | 28 | | 1.15 | 0.90–1.47 | | | |
| Moderate | | | 31 553 | | 590 593 | | | 206 | | 35 | | 1.37 | 1.06–1.76 | | | |
| High | | | 60 092 | | 1 142 380 | | | 427 | | 37 | | 1.53 | 1.21–1.93 | | | |
| *Magnitude of hand-arm vibration (HAV)* | | | | | | | | | | | | | | | | |
| Reference | | | 18 806 | | 307 167 | | | 87 | | 28 | | 1.00 | Ref | | | |
| Low | | | 50 644 | | 933 050 | | | 273 | | 29 | | 1.14 | 0.90–1.46 | | | |
| Acceptable | | | 70 599 | | 1 400 023 | | | 514 | | 37 | | 1.56 | 1.24–1.97 | | | |
| High | | | 15 674 | | 269 869 | | | 89 | | 33 | | 1.26 | 0.94–1.70 | | | |
| *Frequency of impact shocks during HAV* | | | | | | | | | | | | | | | | |
| Reference | | | 18 806 | | 307 167 | | | 87 | | 28 | | 1.00 | Ref | | | |
| Rare | | | 92 879 | | 1 764 447 | | | 605 | | 34 | | 1.37 | 1.09–1.72 | | | |
| Often | | | 44 038 | | 838 495 | | | 271 | | 32 | | 1.35 | 1.06–1.73 | | | |
| *N – number workers; IR – incidence rate per 100 000 person-years; RR – relative risk; CI – confidence interval* | | | | | | | | | | | | | | | | |

| **Table S 5.** Biomechanical risk factors and the incidence rate (IR) and relative risk (RR) for CMC1 OA, M18.3 and M18.5 excluded. RR were adjusted for BMI, smoking, age, and calendar time of surgery. The foremen and white-collar workers were used as reference. | | | | | | |
| --- | --- | --- | --- | --- | --- | --- |
|  | N | person-years | cases | IR | RR | 95% CI |
| *Magnitude of handgrip force* | | | | | | |
| Reference | 30 640 | 486 498 | 130 | 27 | 1.00 | Ref |
| Low | 18 189 | 293 082 | 83 | 28 | 1.01 | 0.72–1.34 |
| Moderate | 78 163 | 1 459 935 | 400 | 27 | 1.21 | 0.99–1.48 |
| High | 110 533 | 1 994 819 | 713 | 36 | 1.53 | 1.27–1.85 |
| *Frequency of pinch grip* | | | | | | |
| Reference | 30 640 | 486 498 | 130 | 27 | 1.00 | Ref |
| Low | 21 111 | 350 423 | 95 | 27 | 1.00 | 0.77–1.31 |
| Moderate | 114 818 | 2 051 374 | 721 | 35 | 1.49 | 1.24–1.80 |
| High | 70 956 | 1 346 040 | 380 | 28 | 1.26 | 1.03–1.55 |
| *Frequency of repetitive wrist flexion and extension work* | | | | | | |
| Reference | 30 640 | 486 498 | 130 | 27 | 1.00 | Ref |
| Low | 73 199 | 1 253 415 | 398 | 32 | 1.29 | 1.05–1.57 |
| Moderate | 51 976 | 981 767 | 297 | 30 | 1.34 | 1.09–1.65 |
| High | 81 710 | 1 512 654 | 501 | 33 | 1.44 | 1.19–1.75 |
| *Frequency of full wrist extension* | | | | | | |
| Reference | 30 640 | 486 498 | 130 | 27 | 1.00 | Ref |
| Low | 99 801 | 1 758 813 | 547 | 31 | 1.31 | 1.08–1.59 |
| Moderate | 107 084 | 1 989 023 | 649 | 33 | 1.41 | 1.16–1.7 |
| High | - | - | - | - | - | - |
| *Frequency of hand-held tool use* | | | | | | |
| Reference | 30 640 | 486 498 | 130 | 27 | 1.00 | Ref |
| Low | 20 527 | 336 589 | 106 | 31 | 1.15 | 0.89–1.49 |
| Moderate | 18 734 | 335 540 | 107 | 32 | 1.34 | 1.04–1.74 |
| High | 167 624 | 3 075 708 | 983 | 32 | 1.39 | 1.16–1.68 |
| *Frequency of heavy lifting* | | | | | | |
| Reference | 30 640 | 486 498 | 130 | 27 | 1.00 | Ref |
| Low | 68 268 | 1 265 786 | 331 | 26 | 1.12 | 0.92–1.38 |
| Moderate | 49 841 | 884 320 | 313 | 35 | 1.47 | 1.19–1.80 |
| High | 88 776 | 1 597 731 | 552 | 35 | 1.49 | 1.23–1.81 |
| *Magnitude of hand-arm vibration (HAV)* | | | | | | |
| Reference | 30 640 | 486 498 | 130 | 27 | 1.00 | Ref |
| Low | 76 291 | 1 332 128 | 383 | 29 | 1.18 | 0.96–1.44 |
| Moderate | 103 355 | 1 980 317 | 673 | 34 | 1.51 | 1.25–1.83 |
| High | 27 239 | 435 391 | 140 | 32 | 1.31 | 1.03–1.66 |
| *Frequency of impact shocks during HAV* | | | | | | |
| Reference | 30 640 | 486 498 | 130 | 27 | 1.00 | - |
| Low | 136 094 | 2 462 319 | 797 | 32 | 1.37 | 1.13–1.65 |
| High | 70 791 | 1 285 518 | 399 | 31 | 1.35 | 1.11–1.65 |
| *N – number workers; IR – incidence rate per 100 000 person-years; RR – relative risk; CI – confidence interval* | | | | | | |


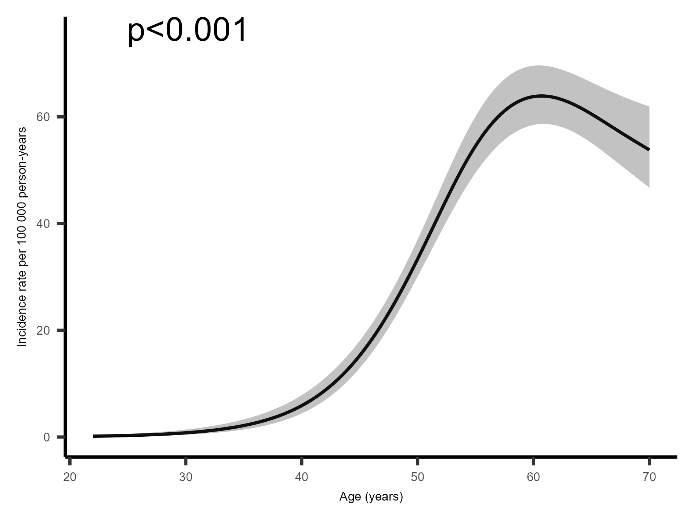


Figure S1. Incidence rate of CMC1 OA per 100 000 person-years by age, adjusted for calendar time. For the visualization, incidence is shown for the year 2006 (median year of follow-up).


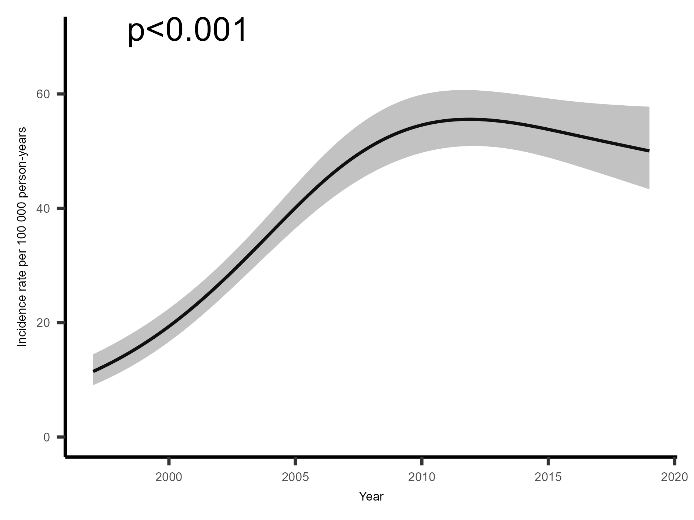


Figure S2. Incidence rate of CMC1 OA per 100 000 person-years by calendar time, adjusted for age. For the visualization, incidence is shown for a 54 year old individual (median age at follow-up).
